# Spontaneous remission of idiopathic minimal change disease in a cat

**DOI:** 10.1177/20551169221131261

**Published:** 2022-11-06

**Authors:** Catherine Broadbridge, Harriet Hall, Katie E McCallum

**Affiliations:** The Queen’s Veterinary School Hospital, University of Cambridge, Cambridge, UK

**Keywords:** Nephrotic syndrome, proteinuria, minimal change disease, effusion, hypoalbuminaemia

## Abstract

**Case summary:**

A 5-year-old female neutered domestic mediumhair cat presented with acute onset hyporexia, lethargy, ascites, hypoalbuminaemia and ventral subcutaneous oedema. Further investigations revealed a bicavitary effusion, myocardial injury, hypercholesterolaemia and concurrent marked proteinuria. A panel of infectious disease tests yielded negative results. Nephrotic syndrome was suspected and renal biopsies were performed. Histopathology and electron microscopy confirmed a diagnosis of minimal change disease (MCD). The patient was successfully managed with benazepril, clopidogrel and a veterinary prescription renal diet. Follow-up two weeks later documented almost complete resolution of the cardiac abnormalities, absence of clinical signs and marked improvement in clinicopathological findings. The hypoalbuminaemia and proteinuria had resolved two months after presentation. At the time of writing, 13 months post-admission, the cat remained asymptomatic with no evidence of disease relapse.

**Relevance and novel information:**

MCD is rarely described in the veterinary literature, with only four cases reported to date. To our knowledge, this report describes the first case of successfully treated MCD-associated nephrotic syndrome in a cat without the use of glucocorticoid treatment.

## Introduction

Minimal change disease (MCD) is a common cause of nephrotic syndrome (NS) in children,^1^ but it has rarely been described in dogs,^2–4^ and only a single feline case report exists in the veterinary literature.^5^ Aetiologies in these cases included drug-induced due to tyrosine kinase inhibitors (n = 2), secondary to infection with *Ehrlichia canis* (n = 6) or idiopathic (n = 1).^2–5^ MCD accounts for up to 90% of cases of NS in children, while in adults it accounts for 10–25% of cases.^6–8^

MCD is a podocytopathy characterised by NS and confirmed histopathologically with evidence of diffuse podocyte foot process effacement, lack of electron-dense deposits on electron microscopy, absence of glomerular lesions by light microscopy and negative or low-level staining for C3 and/or IgM on immunofluorescence microscopy.^7,8^ The disease pathogenesis has not yet been elucidated, but an abnormal T-cell response impairing the glomerular filtration barrier is speculated.^9^ Treatment in humans consists of oral corticosteroids, with gradual tapering of the dose once remission is achieved, although no definitive consensus on dose or duration has been defined in adults.^10^ Spontaneous remission of MCD has been reported in people.^11–16^ Historical prospective, randomised controlled trials documented spontaneous remission of NS in 33–65% of adult patients with MCD.^11–13^ In general, more time was required to achieve this endpoint than those who had glucocorticoid-induced remission.^11–13^

To the best of our knowledge, we report the first documented report of MCD-associated NS that spontaneously remitted without glucocorticoid therapy in a cat.

## Case description

A 5-year-old female neutered domestic mediumhair cat presented at the referring veterinary practice with a history of hyporexia and lethargy. Investigations revealed a mild hypoalbuminaemia (20 g/l; reference interval [RI] 22–40) and hypercholesterolaemia (7.78 mmol/l; RI 1.68–5.81), alongside a mild leukopenia (5.08 × 10^9^/l; RI 5.50–19.50). A feline pancreatic lipase immunoreactivity test (SNAP fPL; IDEXX) was normal. Abdominal ultrasonography findings revealed a large volume of free anechoic peritoneal fluid and a prominent and oedematous pancreas. Abdominocentesis was consistent with a pure transudate. Prior to referral, the ascites had been reported to self-resolve.

On initial presentation to the referral institution the cat weighed 4.4 kg and had a body condition score of 5/9. On thoracic auscultation, the cat was tachypnoeic (respiratory rate 88 breaths/min) with bilaterally harsh bronchovesicular sounds and tachycardic (heart rate 240 beats/min) with a novel grade II/VI parasternal systolic heart murmur. Abdominal palpation documented the presence of ventral abdominal subcutaneous oedema.

Serum biochemistry demonstrated hypoproteinaemia due to hypoalbuminaemia (see Table 1). The mild hypocalcaemia was likely associated with reduced protein-bound calcium. There was a moderate hypercholesterolaemia. Marked elevation of serum amyloid A (SAA) was present, indicating acute inflammation. There was also a moderate elevation in creatine kinase. Haematology documented a mild normocytic normochromic, non-regenerative anaemia (see Table 2). Total thyroxine and preprandial bile acids were normal. Cardiac troponin I was markedly elevated, indicating myocardial injury. Coagulation times documented a mildly elevated activated partial thromboplastin time, but was not deemed clinically significant, while the one-stage prothrombin time was within the RI (Table 2). Blood type was A positive. Urinalysis obtained via cystocentesis revealed inadequately concentrated urine, marked proteinuria (urine protein:creatinine ratio [UPC] 7.57) with inactive sediment analysis (see Table 3). Urine culture was negative.

**Table 1:** Serum biochemistry values, from admission (Day 0) to final follow-up appointment (Day 399)

Parameter	Day 0*	Day 3	Day 8	Day 29	Day 64	Day 241	Day 274	Day 399	RI
Urea (mmol/l)	8.5	NA	10.2	6.9	8.8	8.5	7.9	8.6	5.4–10.7
Creatinine (µmol/l)	91	NA	134	91	128	129	126	120	56–153
Glucose (mmol/l)	5.8	NA	NA	5.6	8.1^†^	5.9^†^	9.8^†^	6.3^†^	3.9–5.8
Total protein (g/l)	53^†^	NA	NA	66	73	74	74	75	56–78
Albumin (g/l)	17^†^	16^†^	22^†^	26	31	34	34	33	25–43
Globulin (g/l)	36	NA	NA	40	42	40	40	42	24–47
Total calcium (mmol/l)	1.9^†^	NA	NA	2.2	2.3	2.4	2.4	2.4	2.0–2.7
Phosphate (mmol/l)	1.4	NA	NA	1.7	1.3	1.4	1.6	1.3	0.9–2.1
ALT (IU/l)	23	NA	NA	23	65^†^	26	29	33	17–62
AST (IU/l)	51	NA	NA	19	36	19	21	23	0–51
ALP (IU/l)	15	NA	NA	26	29	27	25	32	10–93
Bile acids (preprandial; µmol/l)	2	NA	NA	NA	NA	NA	NA	NA	0–12
Cholesterol (mmol/l)	8.1^†^	NA	NA	5.2^†^	5.4^†^	5.4^†^	5.4^†^	6.6^†^	1.7–4.9
CK (IU/l)	1352^†^	NA	NA	231^†^	821^†^	184^†^	182^†^	371^†^	33–168
Lipase (DGGR) (IU/l)	18	NA	NA	15	22^†^	12	13	12	0–19
SAA (µg/ml)	65.2^†^	NA	<0.3	<0.3	<0.3	<0.3	<0.3	<0.3	0–0.5
Total T4 (nmol/l)	19	NA	NA	NA	NA	NA	NA	NA	7–45
Troponin I (ng/ml)	9.384^†^	NA	NA	0.144^†^	NA	0.039	NA	NA	0–0.04

* Initial presentation

† Abnormal value

RI = reference interval; NA = not applicable; ALT = alanine transaminase; AST = aspartate transaminase; ALP = alkaline phosphatase; CK = creatine kinase; DGGR = 1,2-o-dilauryl-rac-glycero-3-glutaric acid-(6’-methylresorufin) ester; SAA = serum amyloid A; T4 = thyroxine

**Table 2:** Haematology values, from admission (Day 0) to final follow-up appointment (Day 399)

Parameter	Day 0*	Day 3	Day 8	Day 29	Day 64	Day 241	Day 274	Day 399	RI
WBCs (×10^9^/l)	5.68	NA	4.77^†^	5.11^†^	NA	5.38^†^	5.70	4.43^†^	5.5–19.5
Neutrophils (×10^9^/l)	3.33	NA	3.08	3.67	NA	2.92	4.05	3.28	2.5–12.5
Lymphocytes (×10^9^/l)	1.5	NA	1.2^†^	0.8^†^	NA	1.6	1.1^†^	0.5^†^	1.5–7.0
Monocytes (×10^9^/l)	0.39	NA	0.22	0.15	NA	0.23	0.20	0.09	0–1.5
Eosinophils (×10^9^/l)	0.42	NA	0.29	0.45	NA	0.63	0.36	0.44	0–1.5
Basophils (×10^9^/l)	0.02	NA	0	0	NA	0.02	0.00	0.09	0–0.5
HCT (%)	21.3^†^	NA	22.1^†^	26.7	NA	35.6	35.1	33.5	26–45
MCV (fl)	44.5	NA	51.5	48.1	NA	42.1	42.6	43.1	39–55
MCHC (g/dl)	35.7	NA	33.9	33.0	NA	37.1^†^	36.2^†^	37.3^†^	30–36
RDW (%)	14.9^†^	NA	26.5^†^	14.4	NA	17.3^†^	17.6^†^	17.0	11.6–14.8
Platelets (×10^9^/l)	94^†^	NA	156^†^	337	NA	46^†^ (platelet clumps and macroplatelets)	205	54^†^ (platelet clumps and macroplatelets)	200–800
PCV (%)	22^†^	15^†^	22^†^	28	NA	38	38	35	26–45
PP (g/l)	54^†^	50^†^	68	68	NA	74	76	76	60–80
Reticulocytes (×10^9^/l)	15	NA	162^†^	20	NA	14	27	9	0–60
OSPT (s)	13.3^†^	NA	NA	NA	NA	NA	NA	NA	7–11
aPTT (s)	12.9	NA	NA	NA	NA	NA	NA	NA	10–15

* Initial presentation

† Abnormal value

RI = reference interval; WBCs = white blood cells; NA = not applicable; HCT = haematocrit; MCV = mean cell volume; MCHC = mean cell haemoglobin concentration; RDW = red cell distribution width; PCV = packed cell volume; PP = plasma protein; OSPT = one-stage prothrombin time; aPTT = activated partial thromboplastin time

**Table 3:** Urinalysis via cystocentesis sample collection, from admission (Day 0) to final follow-up appointment (Day 399)

Parameter	Day 0*	Day 3	Day 8	Day 29	Day 64	Day 241	Day 274	Day 399	RI
UPC	7.57^†^	2.84^†^	3.83^†^	0.61^†^	0.13	0.10	0.09	0.09	0–0.2
USG	1.028	1.017^†^	1.027	1.010^†^	1.016^†^	1.030	1.032	1.016^†^	
pH	7.0	8.0	6.0	7.0	7.0	6.0	6.0	6.5	
Glucose dipstick	Negative	Negative	Negative	Negative	Negative	Negative	Negative	Negative	
Bilirubin dipstick	Negative	Negative	1+^†^	Negative	1+^†^	Negative	Negative	Negative	
Erythrocytes/Hb dipstick	2+^†^	Trace^†^	4+^†^	Negative	Negative	4+^†^	4+^†^	Negative	
WBC microscopy (cells/HPF)	1^†^	0	0	0	0	1	0	0	0–5
RBC microscopy (cells/HPF)	5^†^	0	>200^†^	0	2	65^†^	60^†^	0	0–5

* Initial presentation

† Abnormal value

RI = reference interval; UPC = urine protein:creatinine ratio; USG = urine specific gravity; Hb = haemoglobin; WBC = white blood cell; HPF = high-power field; RBC = red blood cell

### Infectious disease testing

Feline coronavirus (FCoV), *Bartonella henselae* and *Toxoplasma gondii* serology were negative. FCoV RT-PCR and *B henselae* PCR from pleural fluid and blood, respectively, were negative. Additionally, a feline leukaemia virus antigen/feline immunodeficiency virus antibody test (SNAP FIV/FeLV Combo Test; IDEXX) was negative.

### Imaging findings

**Figure 1:**
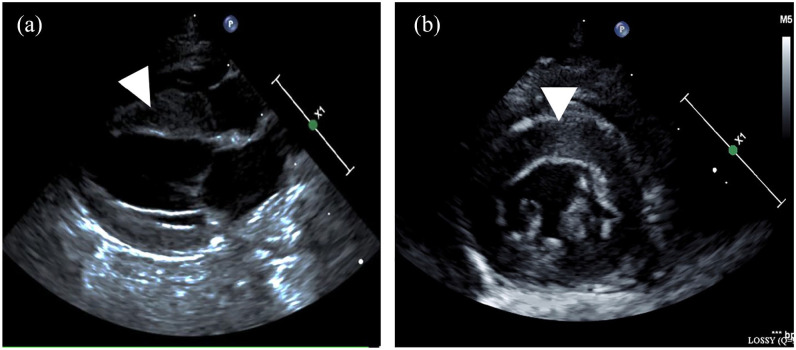
Two-dimensional echocardiographic images from the (a) right parasternal long-axis view and (b) short-axis view demonstrating a hypokinetic hypertrophied interventricular septum, with septal thickening up to 7.1 mm (reference interval <6)

Echocardiography revealed a hypokinetic hypertrophied interventricular septum (interventricular septal end diastole thickness 7.1 mm; RI <6) despite a normal left ventricular (LV) free wall thickness (5.2 mm; RI <6) and LV free-wall motion (LV fractional shortening 33%; RI >30%) (Figure 1). The patient had a normal left atrial (LA) size (LA:Ao 1.4, RI < 1.6), with normal LA systolic function (LA fractional shortening 36%; RI >20%). These findings were not typical for a hypertrophic cardiomyopathy (HCM) phenotype but possibly associated with an HCM phenocopy, such as one that develops with infiltrative diseases.

**Figure 2:**
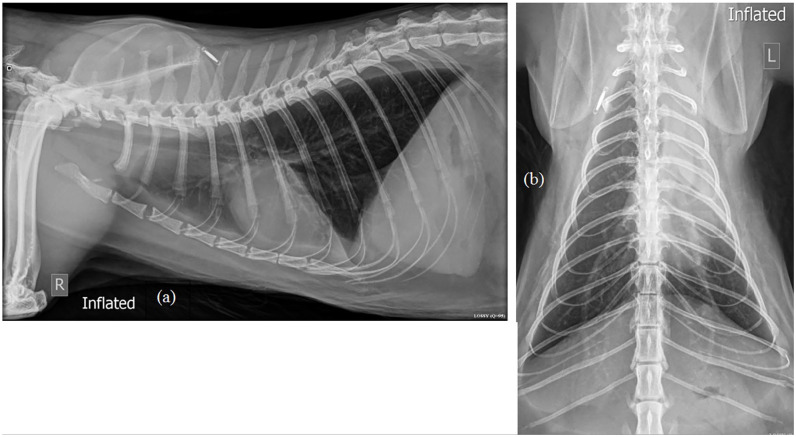
Thoracic radiographs, (a) right lateral and (b) dorsoventral views, demonstrating a homogeneous soft tissue opacity in the region of the left cranial lung lobe, with an air bronchogram present within the lobe and a mediastinal shift to the left. A ventral fluid line and pleural fissure lines are present, indicating a small volume of pleural effusion. In the remainder of the lung fields a mild bronchial pattern is noted

Thoracic radiographs documented collapse of the left cranial lung lobe, likely due to atelectasis, bronchial plugging, a previous thrombus or a previously large volume of pleural effusion. The ventral lung lobes were retracted from the thoracic wall, with a homogeneous ventral fluid opacity with pleural fissure lines present (Figure 2). The remainder of the lung fields had a mild bronchial pattern consistent with lower airway disease or age-related fibrosis. Thoracic ultrasonography confirmed the presence of consolidated left and right ventral cranial lung lobes and bilateral anechoic pleural effusion.

Ultrasonographically, there was a small volume of anechoic peritoneal fluid and oedematous mesentery in the region of the ileocaecal colic junction. Anechoic fluid was interspersed throughout the ventral abdominal subcutaneous tissues, consistent with ventral abdominal subcutaneous oedema.

Cytology of the pleural fluid was consistent with a pure transudate, with nucleated cells comprised predominantly of a population of macrophages and lower numbers of small lymphocytes and occasional non-degenerate neutrophils.

### Renal cytology and histology findings

**Figure 3:**
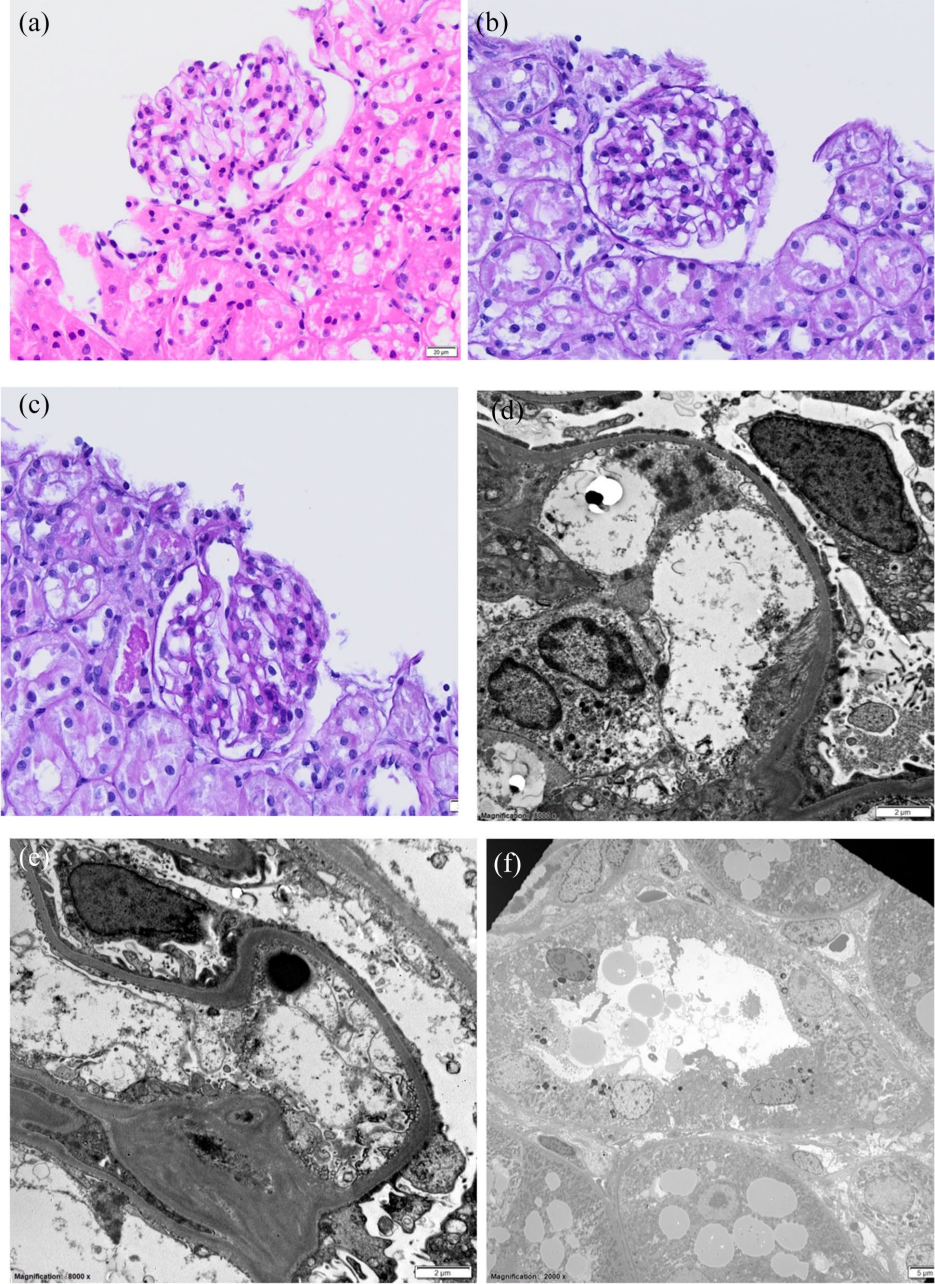
(a–c) Light microscopy images. (a) Haematoxylin and eosin and (b,c) periodic acid–Schiff, of unremarkable glomeruli and evidence of mild acute tubular epithelial degeneration with a few intratubular protein casts. (d–f) Electron microscopy images revealing severe widespread effacement of podocyte foot processes and no evidence of electron-dense deposits in the capillary loops or mesangial zones. (f) Tubular loss of the apical brush border

Elective surgical Tru-Cut renal biopsies (Tru-Cut Biopsy Device, 18 G) and fine-needle aspirates were obtained for cytology, histopathology, electron microscopy, light microscopy and immunofluorescence. Renal cytology revealed well-differentiated tubular epithelial cells. Light microscopy using haematoxylin and eosin staining (Figure 3a), periodic acid–Schiff staining (Figure 3b,c), Masson’s trichome staining and Jones methenamine silver staining documented 13 normal glomeruli with mild acute renal tubular epithelial degeneration and few intratubular protein casts. However, electron microscopy demonstrated two glomeruli with severe widespread effacement of podocyte foot processes and swelling of podocyte cytoplasm (Figure 3). No electron-dense deposits were identified along the capillary loops or in mesangial zones. There were four glomeruli available for immunofluorescence, which documented trace to weak staining of IgG, IgA, lambda light chains and IgM. These lesions indicate that the podocyte lineage is injured, warranting a diagnosis of minimal change disease.

### Outcome

Repeated urinalysis (Table 3), serum biochemistry (Table 1) and PCV (Table 2) on day 3 revealed an improvement in proteinuria (UPC 2.84) and worsening of anaemia and hypoalbuminaemia. Repeat thoracic radiographs revealed improved inflation in the left cranial lung lobe and reduction in the volume of pleural effusion. Owing to the clinical improvement and fear-aggressive temperament, the cat was discharged with benazepril (2.5 mg q24h PO [Benazecare; Animalcare]), clopidogrel (18.75 mg q24h PO [Plavix; Sanofi-Aventis]), buprenorphine (0.02 mg/kg q8h transmucosally [Vetergesic; Ceva Animal Health]) and maropitant (8 mg q24h PO [Cerenia; Zoetis]).

One week after discharge, there was resolution of the tachypnoea, inappetence and lethargy. Haematology revealed a regenerative anaemia (Table 2). Serum biochemistry revealed an improved hypoalbuminaemia and resolution of the previously elevated SAA (Table 1). Urinalysis documented persistent proteinuria and marked haematuria, likely as a sequelae to the recent renal biopsy (Table 3). Repeat echocardiography was consistent with mild interventricular septum hypertrophy but with normal systolic function and novel trace amounts of pericardial effusion, suspected to be secondary to an ongoing inflammatory process. Thoracic radiography was unremarkable. Focused abdominal ultrasonography identified trace anechoic peritoneal fluid.

The patient was discharged with an increased dose of benazepril (2.5 mg q12h PO) and a protein-restricted renal diet (Royal Canin Feline Renal Adult).

At follow-up two weeks later (day 29), there was resolution of the hypoalbuminaemia (Table 1) and anaemia (Table 2), a reduction in cholesterol and troponin I (Table 1), and a marked improvement in UPC (Table 3).

On day 64, there was resolution of proteinuria and the benazepril was gradually tapered based on serial urinalyses. At the last follow-up visit (13 months following initial presentation), the patient was receiving 1.25 mg q24h PO of benazepril and a prescription renal diet with no evidence of relapse; therefore, it was advised for the benazepril to be discontinued with ongoing close monitoring of clinical and clinicopathological changes.

## Discussion

To our knowledge, only a single feline case of MCD has been previously described in the veterinary literature, presumed to be secondary to the use of imatinib mesylate, a tyrosine kinase inhibitor.^5^ It was reported that discontinuation of imatinib mesylate, alongside a tapering dose of glucocorticoids, led to clinical resolution but only partial resolution of proteinuria. This case report represents the first feline case of NS due to MCD that underwent suspected spontaneous resolution.

MCD is categorised as either primary (idiopathic) or secondary to a putative cause. Secondary forms of MCD are recognised in people, although the underlying pathophysiological mechanisms have not yet been elucidated. Hodgkin’s lymphoma, non-Hodgkin’s lymphoma and thymoma are the most frequently reported paraneoplastic processes associated with MCD.^7,17–19^ Certain drugs are presumed to cause either a hypersensitivity reaction or direct toxic effect to glomerular epithelial cells, leading to suspected drug-induced MCD, namely non-steroidal anti-inflammatory drugs and antimicrobials, although several other drugs classes have been reported.^18^ While sporadic reports of infectious agent-mediated MCD also exist, a direct causal relationship has not been established, although the NS inherently confers an increased infection risk.^18^ Lastly, multiple reports suggest a link between IgE-mediated hypersensitivity allergic reactions and the development of MCD in humans.^18^ Two previously published veterinary case reports attribute the use of tyrosine kinase inhibitors with the induction of MCD, given that remission was achieved once the drug was discontinued.^2,5^ Additionally, transient MCD has been described in six dogs with experimentally induced E. canis, in which proteinuria self-resolved once the infection was cleared after 10 weeks.^3^ In our report, a detailed owner history was acquired, the previous medical records were analysed, and thoracic and abdominal imaging and a comprehensive infectious disease testing panel were performed that revealed no obvious drug, infectious, allergic, immune-mediated or neoplastic cause. As a result of excluding most secondary causes of MCD, idiopathic MCD was deemed most probable.

Classically, owing to the high prevalence of MCD in children <10 years of age with NS, a renal biopsy is not indicated unless unexpected laboratory or clinical findings exist. In adults, MCD accounted for only 4.1% of renal biopsy samples in one centre from 2006 to 2015.^1^ Therefore, biopsies are an invaluable diagnostic and prognostic tool in adult MCD. In line with the International Renal Interest Society (IRIS) consensus recommendations for the diagnosis of canine glomerular disease, renal biopsies were taken in our patient given the high-magnitude persistent proteinuria (UPC ⩾3.5) and evidence of NS.^20^ This was pursued to provide a definitive diagnosis and to guide therapeutic decision-making, particularly focusing on immunostaining techniques to identify an immunopathogenesis that may require immunosuppressive therapy. Repeat renal biopsies would have been beneficial to understand whether histopathological improvement coincided with clinical and clinicopathological resolution, and whether there was evidence of focal segmental glomerulosclerosis (FSGS) development, a postulated sequelae of MCD in people.^21,22^ However, owing to the associated risks, they were not repeated, representing a limitation of this report.

NS, in particular rapid oedema development, is the most common presenting symptom of MCD in people.^7^ NS is associated with significant morbidity and mortality, and, if left untreated, can cause thromboembolic events, end-stage kidney disease, cardiovascular disease and death. Owing to these sequelae, current recommendations are to treat MCD with first-line glucocorticoids. Patients with NS are classified as either steroid-sensitive or steroid-resistant.^6^ The latest systematic reviews recommend an initial oral prednisolone dose of 1 mg/kg/day in adults and 2 mg/kg/day in children for a minimum of 4–8 weeks until complete remission (defined by daily urine protein excretion of <0.3 g/day, UPC <0.3, or trace or negative results on repeat urine protein dipstick) has been achieved, followed by gradual tapering over 6 months.^21,23,24^ Complete remission occurs in >90% of steroid-responsive people; however, adults take longer to achieve remission with steroid treatment.^24,25^

Prednisolone has well-established anti-inflammatory and immunosuppressive beneficial effects, including a direct protective effect on podocytes.^26^ However long-term glucocorticoid exposure, particularly in those with relapsing disease, will often require high doses and repeated courses leading to cumulative effects associated with significant adverse effects. Glucocorticoid adverse effects include weight gain, immunosuppression, gastrointestinal ulceration and diabetes mellitus. Use of prednisolone was associated with increased mortality in adults with MCD, with a mortality rate of 28% and 19% in the prednisolone-treated and control groups, respectively, with the major cause of mortality in the prednisolone group attributed to iatrogenic steroid complications.^11^

The standard therapeutic considerations in the management of proteinuria remain unchanged, regardless of the type of glomerular disease, and are centred around inhibition of the renin–angiotensin–aldosterone system with an angiotensin-converting enzyme inhibitor as a first-line therapeutic and dietary modification. However, this information is extrapolated from canine guidelines.^27^ Unfortunately, owing to patient temperament, blood pressure was not measured. A protein-restricted diet was implemented, as there is evidence to suggest restricted protein intake reduces proteinuria, in turn slowing progression of proteinuric kidney disease.^27^ Thromboembolic events are reported to occur in 13% of dogs affected by protein-losing glomerular disease, leading to fatal complications in 22% of dogs; therefore, prophylaxis with antithrombotics is recommended.^28,29^ In people and dogs, arterial thromboembolism predominates over venous thromboembolism, and antiplatelet drugs reduce the risk of thrombosis in this population.^30,31^ Therefore, extrapolation of this information, in combination with a recent study evaluating thromboprophylaxis in feline arterial thromboembolism, led to prophylactic clopidogrel treatment in this case.^29,32^

Spontaneous remission of MCD has been reported in adult humans.^11–16^ Remission rates of 33–65% have been reported, although a longer duration of time is usually required to achieve this endpoint vs those treated with corticosteroids.^11–13^ Two of the previous reports of dogs that developed MCD as a result of secondary causes also spontaneously remitted without corticosteroids once the inciting cause was treated or discontinued.^2,3^ Arguably, the side effect profile of corticosteroids, in combination with the potential for spontaneous remission, led to the omission of corticosteroid treatment in our patient.

Acute kidney injury (AKI) is reported in 25% of patients with MCD.^21^ Although our patient was non-azotaemic, it demonstrated inadequate urine-concentrating ability and renal histopathology documented acute tubular degeneration, which may suggest a degree of AKI, notably an IRIS grade 1 AKI.^33^ Proposed mechanisms behind AKI in MCD indicate that the exposure of proximal tubular cells to albumin can result in endoplasmic reticulum stress, induction of apoptosis, tubular chemokine and cytokine expression, activation of the complement cascade, leading to renal interstitial oedema and ischaemic tubular injury.^21^ Usually, the AKI is reversible, although in some cases residual renal impairment may occur.^21^

Only 4% of steroid-sensitive people progress to develop end-stage kidney disease up to 6 years post-MCD diagnosis.^21^ Some studies propose a progression or overlap of MCD steroid-resistant individuals with FSGS, as demonstrated by repeated renal biopsies.^21,22,34^ This is suspected to occur as a result of podocytotoxic factors, such as the persistence of nephrotic range proteinuria and glomerular hypertension.^21,22^ Given the infrequency of diagnosis of MCD in the veterinary population and overlap in histopathological/morphological features with FSGS, such as diffuse podocyte foot process effacement and absence of electron-dense immune deposits, we cannot entirely exclude idiopathic FSGS as a cause of NS in our patient. This represents a limitation of this report, as – ideally – a greater number of glomeruli would need to be sampled to reasonably exclude FSGS due to its focal nature. Repeat renal biopsies could be considered in our patient, if relapse and/or reduced response to steroids occurs, to evaluate for progression to FSGS.

The aetiology of the cardiac abnormalities in our patient remains unknown; however, given the serial improvement in cardiac troponin I and echocardiographic changes, myocardial injury secondary to transient myocarditis was deemed most likely. There is often an antecedent infection that triggers onset of MCD in people.^7^ The underlying cause could be attributed to an infectious, inflammatory or immune-mediated entity. Viruses are the most common cause of transient myocarditis in humans, and despite considerable infectious disease testing in our patient, an endomyocardial biopsy would be the gold-standard test for diagnosis of cardiotropic viruses.^35^ Alternatively, a systemic/generalised vasculitis was also postulated as a differential diagnosis, which could manifest as myocarditis or pericarditis, although cardiac involvement in primary systemic vasculitides occurs in <10% of humans.^36^ As a result, the above could not be excluded as a potential trigger of MCD, although an idiopathic MCD was considered most likely, due to spontaneous remission and lack of relapse.

Despite 13 months of follow-up with a favourable outcome, future relapse of MCD-associated NS in our patient will remain unknown. Two thirds of steroid-sensitive adults will relapse, while approximately 80% of children will experience relapse.^25,37,38^ Second-line immunosuppressive medications are indicated in patients that frequently relapse, are steroid-dependent or have intolerable steroid adverse effects.^7,10^ Of the few published veterinary case reports of MCD, all surviving patients either completely remitted without relapse or clinically improved with only partial resolution of proteinuria during the reported follow-up period.^2–5^

## Conclusions

This report documents a novel case of spontaneously remitting idiopathic MCD-associated NS in a cat, without the use of glucocorticoids, a first-line therapeutic in human medicine. The significance and aetiology of the cardiac abnormalities remains unknown; however, a transient myocarditis was deemed most probable, although the relevance of its association with the podocytopathy remains contentious.
